# Integrin α5 triggers the metastatic potential in renal cell carcinoma

**DOI:** 10.18632/oncotarget.22501

**Published:** 2017-11-18

**Authors:** Ines Breuksch, Franz Prosinger, Fabian Baehr, Franz-Peter Engelhardt, Heide-Katharina Bauer, Joachim W. Thüroff, Anne-Sophie Heimes, Annette Hasenburg, Dirk Prawitt, Walburgis Brenner

**Affiliations:** ^1^ Department of Gynecology, Johannes Gutenberg University Medical Center, 55131 Mainz, Germany; ^2^ Department of Urology, Johannes Gutenberg University Medical Center, 55131 Mainz, Germany; ^3^ Department of Pediatrics, Johannes Gutenberg University Medical Center, 55131 Mainz, Germany

**Keywords:** integrin α5, renal cell carcinoma, metastasis, clear cell RCC, cell adhesion molecule

## Abstract

The therapy of advanced renal cell carcinoma (RCC) is still a major challenge. To intervene therapeutically a deeper comprehension of the particular steps of metastasis is necessary. In this context membrane bound receptors like integrins play a decisive role. We analyzed the integrin α5 expression in 141 clear cell RCC patients by Western blot. Patients with RCC expressed a significant higher level of integrin α5 in tumor than in normal tissue. The integrin α5 expression correlated with tumor grade, the development of distant metastases within five years after tumor nephrectomy and reduced survival. The RCC cell lines Caki-1 and CCF-RC1, which highly express integrin α5, were treated with fibronectin in combination with or without an inhibiting anti-integrin α5 antibody. Afterwards the migration, adhesion, viability and prominent signaling molecules were analyzed. Both cell lines showed a significant reduced migration potential as well as a decreased adhesion potential to fibronectin after treatment with an integrin α5 blocking antibody. A contribution of the AKT and ERK1/2 signaling pathways could be demonstrated. The results indicate integrin α5 as a potent marker to discriminate patients’ tumor prognosis. Consequently the integrin subunit α5 can be considered as a target for individual therapy of advanced RCC.

## INTRODUCTION

Integrins are a major family of transmembrane heterodimer glycoproteins. They mediate several focal adhesion contacts between cells and extracellular matrix (ECM) components, therefore inducing cell processes like migration, adhesion, proliferation and apoptosis [1, 2]. The 24 integrin receptors are composed of one of the 18 alpha and one of the 8 beta subunits, each subunit with an extracellular domain, a transmembrane region and a short cytoplasmic tail [3]. Most integrins bind their ligands via the arginine-glycine-aspartic acid (RDG) sequence and consequently recruit different signaling kinases like the focal adhesion kinase (FAK) or SRC (Rous sarcoma oncogene cellular homologue) homology 2 containing-protein (SHC), then activate pathways like the mitogen activated protein kinase (MAPK) signaling cascade or the phosphatidylinositol-4,5-bisphosphate 3-kinase (PI3K)/AKT8 virus oncogene cellular homolog (AKT) signaling cascade and as a result regulate cellular processes [4, 5]. The subunit α5 forms together with β1 a heterodimer receptor, that mainly binds to and is activated by the ECM component fibronectin [3].

Because of its broad range of regulative opportunities in cellular mechanisms integrins are able to induce tumor processes. In colon carcinoma and basal-like breast cancer increased integrin α5 is associated with tumor progression and metastasis [6–8]. Murillo *et al.* showed that colon cancer cells had a reduced cell attachment and an increase in apoptosis after inhibiting integrin α5 [9]. Other group figured out that integrin α5 triggers the activity of P-selectin and human carcinoembryonic antigen (CEA), thus promoting tumor progression [10, 11]. The pathologic increase of integrin α5 has been demonstrated to be the consequence of known oncogenic factors. So is the oncogene ERBB2 able to increase the expression of integrin α5, thus enhancing tumor invasion and survival in breast cancer [12]. One explanatory approach for the effects of the invasive role of this subunit is its ability to modulate several matrix metalloproteinases (MMP) [13, 14]. Another factor, angiopoetin-2, is also capable to trigger the integrin α5 expression, leading to increased adhesion and migration of breast cancer cells [15]. Huang *et al.* suggested a participation of the reductase AKR1B10 which promotes breast cancer metastasis via integrin α5 [16]. In addition the transcription factor RUNX2 may play a role in these processes [17]. Furthermore, an increased expression of integrin α5 is associated with a worse outcome in cancer entities like non-small cell lung cancer, high-grade glioma or ovarian carcinoma [18–21]. In ovarian cancer cells Gong *et al.* have shown that the miR-17 inhibits peritoneal metastasis via an integrin α5 dependent cascade [22]. Likewise the loss of E-cadherin induces an integrin α5 dependent spread of tumor cells in ovarian cancer [23]. Integrin α5 can additionally induce the oncogene cMet, promoting tumor invasion and metastasis [24]. In renal cell carcinoma (RCC) Hase *et al.* have shown that the LOX-like protein (LOXL2) promotes tumor progression by regulating integrin α5 levels [25]. In a previous study we demonstrated that integrin α5 participates in bone metastasis processes of RCC [26]. Both studies suggest a progression promoting role of integrin α5 in kidney cancer, although details of this function are still unknown. In the presented study we analyzed the expression of integrin α5 in a cohort of RCC patients and subsequently investigated its role in important cellular processes of metastasis *in vitro*.

## RESULTS

### Integrin α5 protein expression in patients with clear cell RCC

**Figure 1:**
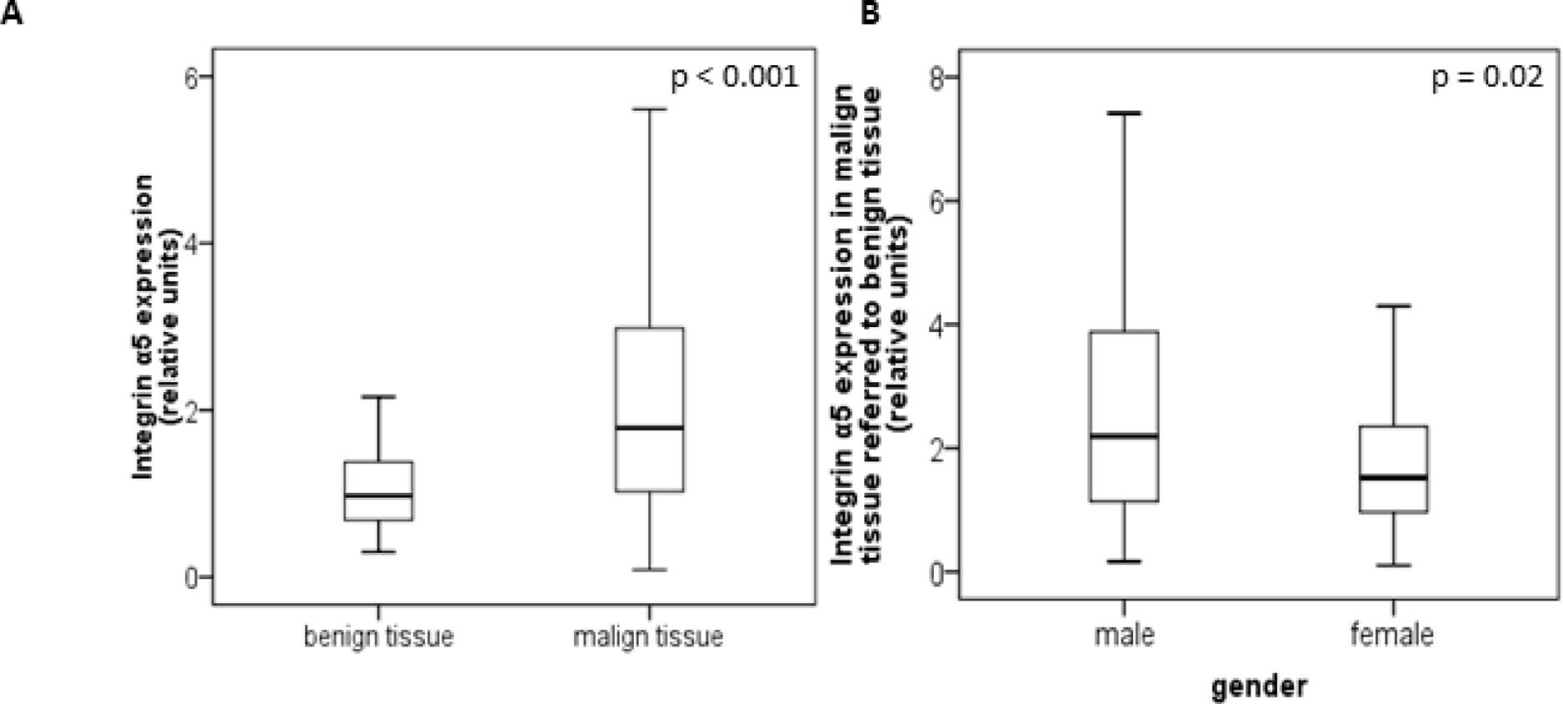
Protein level of integrin α5 in benign and malign renal tissue of 141 patients with clear cell RCC (**A**) Integrin α5 expression in benign and the corresponding malign renal tissue. Tumor tissue showed a significant higher integrin α5 expression. Significance was calculated by a Wilcoxon signed-rank test, *p* < 0.001. (**B)** Gender-specific integrin α5 expression of malign referred to corresponding benign tissue. Male patients show a significant higher integrin α5 expression than female patients. The box-plots show the median and 25% and 75% percentiles of integrin α5 expression level determined by Western blot analysis. Significance was calculated by a Mann–Whitney *U*-test, *p* = 0.02.

**Figure 2:**
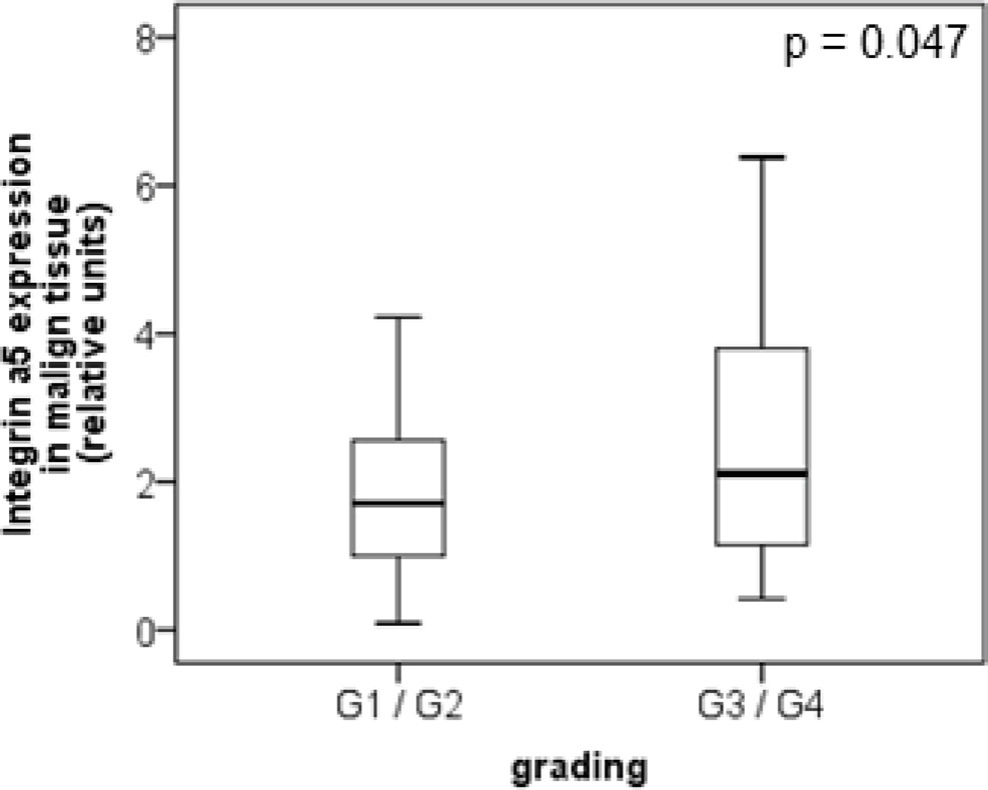
Protein level of integrin α5 in clear cell RCC patients depending on tumor grade In low-grade tumors (G1 and G2, *n* = 87) integrin α5 was significantly lower expressed than in high-grade tumors (G3 and G4, *n* = 54). The box-plots show the median expression values with the 25% and 75% percentiles of integrin α5 expression level determined by Western blot analysis. Significance was calculated by a Mann–Whitney *U*-test, *p* < 0.05.

**Figure 3:**
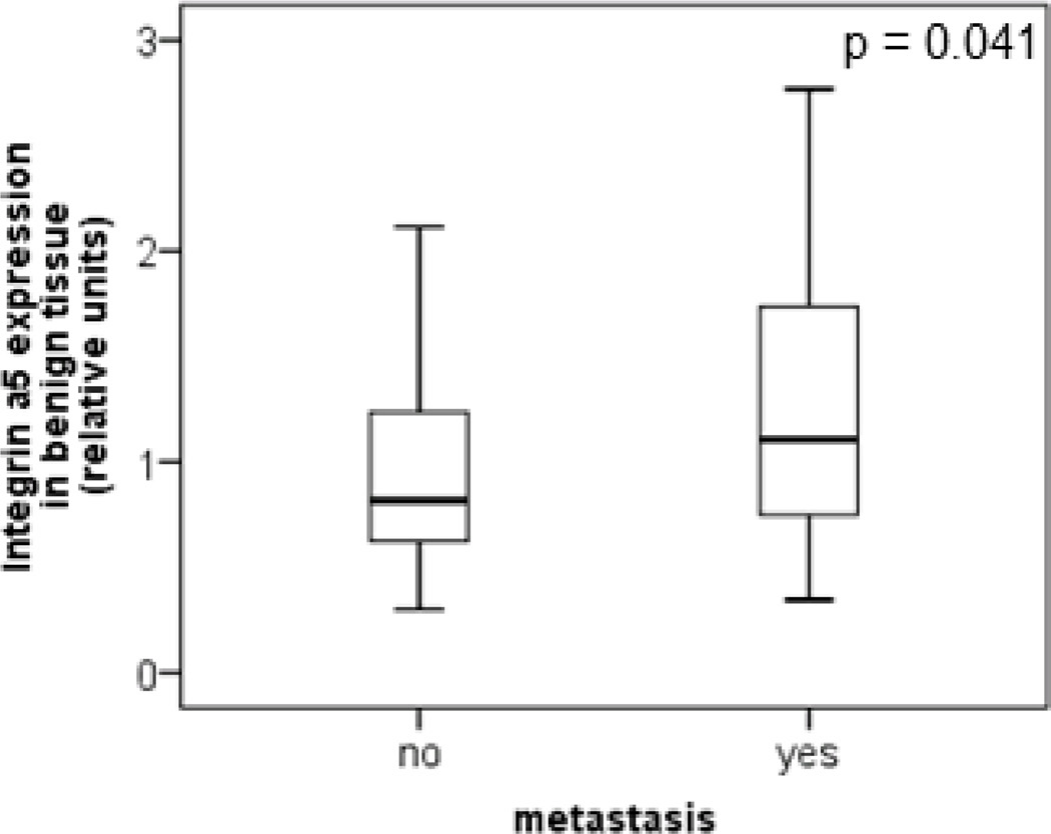
Protein level of integrin α5 in clear cell RCC patients depending on the metastatic status Integrin α5 expression in benign renal tissue of patients who developed metastases within five years after nephrectomy was significantly higher than in patients without metastases. The box-plots show the median expression values and the 25% and 75% percentiles of integrin α5 expression level determined by Western blot analysis. Significance was calculated by a Mann–Whitney *U*-test, *p* < 0.05.

**Figure 4:**
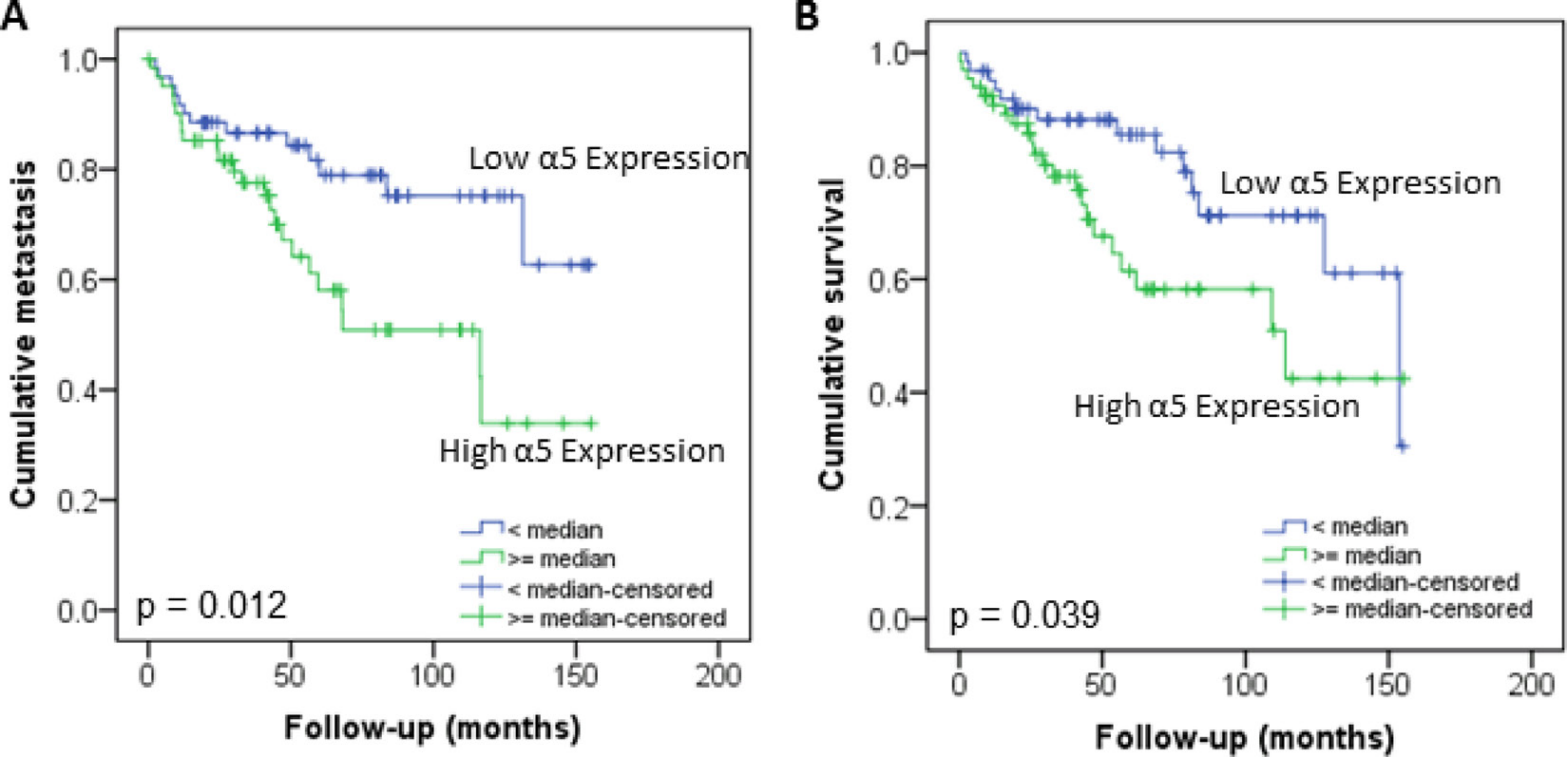
Influence of integrin α5 protein level in normal renal tissue of RCC patients on development of metastasis (**A**) and overall survival (**B**). The Kaplan–Meier curves show a significant lower metastatic rate and higher survival of patients with low integrin α5 expression value in benign renal tissue. Significance was calculated by a Log Rank test, *p* < 0.05.

The protein level of integrin α5 subunit was determined in RCC tumors of a cohort of 141 patients with clear cell RCC (ccRCC). The protein expression of integrin α5 in RCC tissue (median 1.74) was significantly (p < 0.001) higher than in the corresponding normal renal tissue (median 0.95, Figure 1A). Male patients had a significantly higher expression (median 2.19) than female patients (median 1.52, p = 0.02, Figure 1B). The correlation of the integrin α5 expression level in RCC tissue to several prognostic factors of the patients (Table 1) showed a significantly higher integrin α5 level in G3/G4 tumors than in G1/G2 tumors with a median of 2.11 and 1.71, respectively (p = 0.047, Figure 2). The integrin α5 expression in benign renal tissue of RCC patients differed depending on the metastatic status (Figure 3). Patients who developed distant metastases five years after nephrectomy had a significantly higher integrin α5 value (median 1.45 versus 0.82, respectively, p = 0.041). Furthermore, in the group of patients with metastases (n = 124) the integrin α5 expression in benign renal tissue correlated with the development of metastases (p = 0.012, Figure 4A) and a shorter overall survival (p = 0.039, Figure 4B). However, integrin α5 did not turn out as an independent predictor for metastasis, determined by a Cox regression using grading, T-stage and tumor size for covariates.

**Table 1:** Patients‘ data

		frequency
**gender**	male	84
	female	57
**pT-stage**	1	73
	2	21
	3	45
	4	2
**grading**	1	20
	2	67
	3	49
	4	5
**M-stage – 5 years after diagnosis**	Yes	34
	No	90
	Unknown	17
**age (years)**	Median	64.9
**Follow-up (months)**	Median	47.8
	Min	0.3
	Max	155.2

Overview of the composition of the analyzed patient‘s cohort.

### Impact of integrin α5 on chemotactic migration, cell adhesion and viability *in vitro*

**Figure 5:**
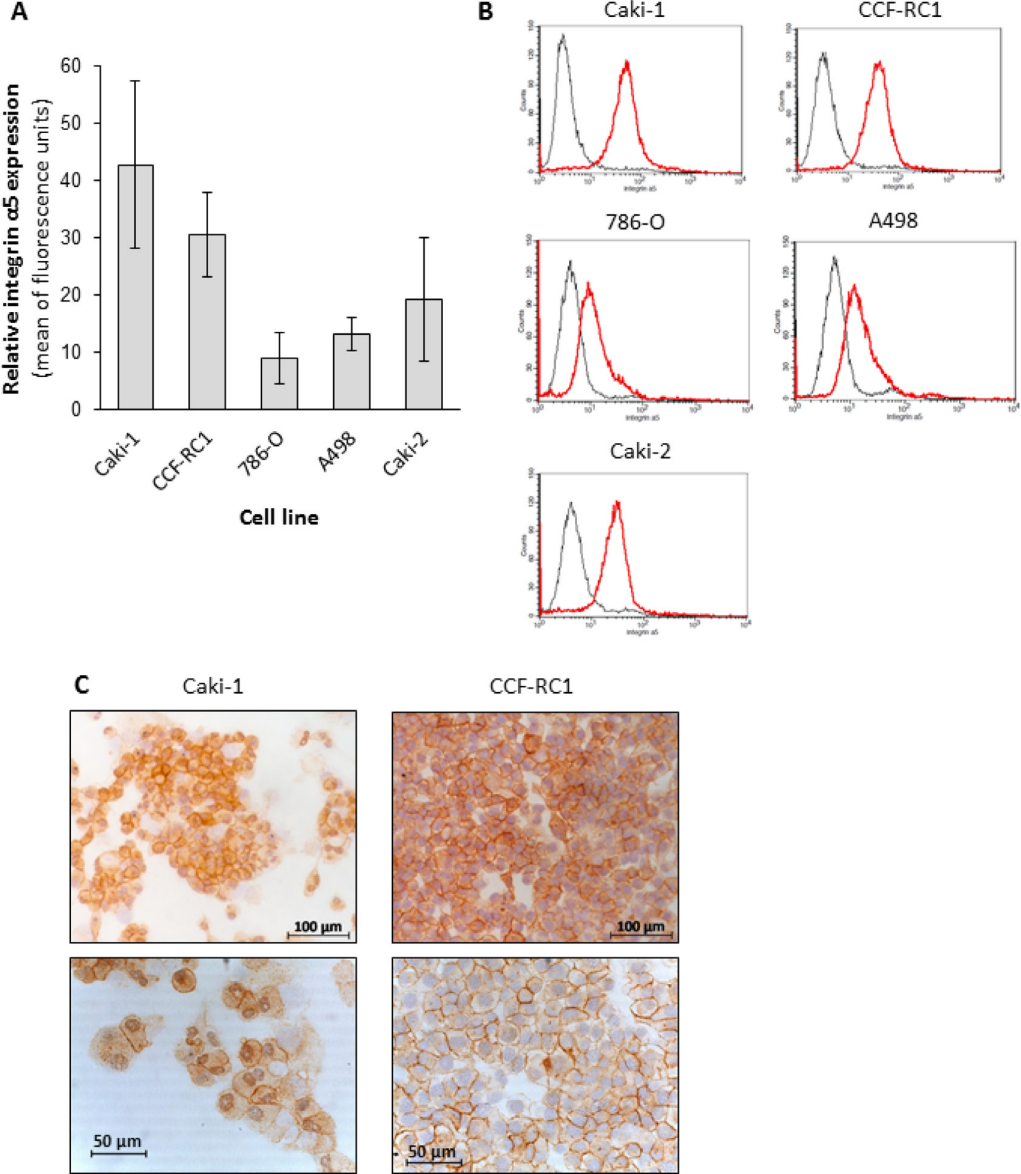
Protein level of integrin α5 in renal carcinoma cell lines The flow cytometric analyses of five ccRCC cell lines identified the cell lines CCF-RC1 and Caki-1 as those with the highest integrin α5 expression (**A, B**). The histograms showed an integrin α5 expression (red graph) comparing to isotype control (grey graph) in all cell lines (B). In an immuno-histochemical staining the integrin α5 expression in these cells was localized at the cell membrane (**C**).

**Figure 6:**
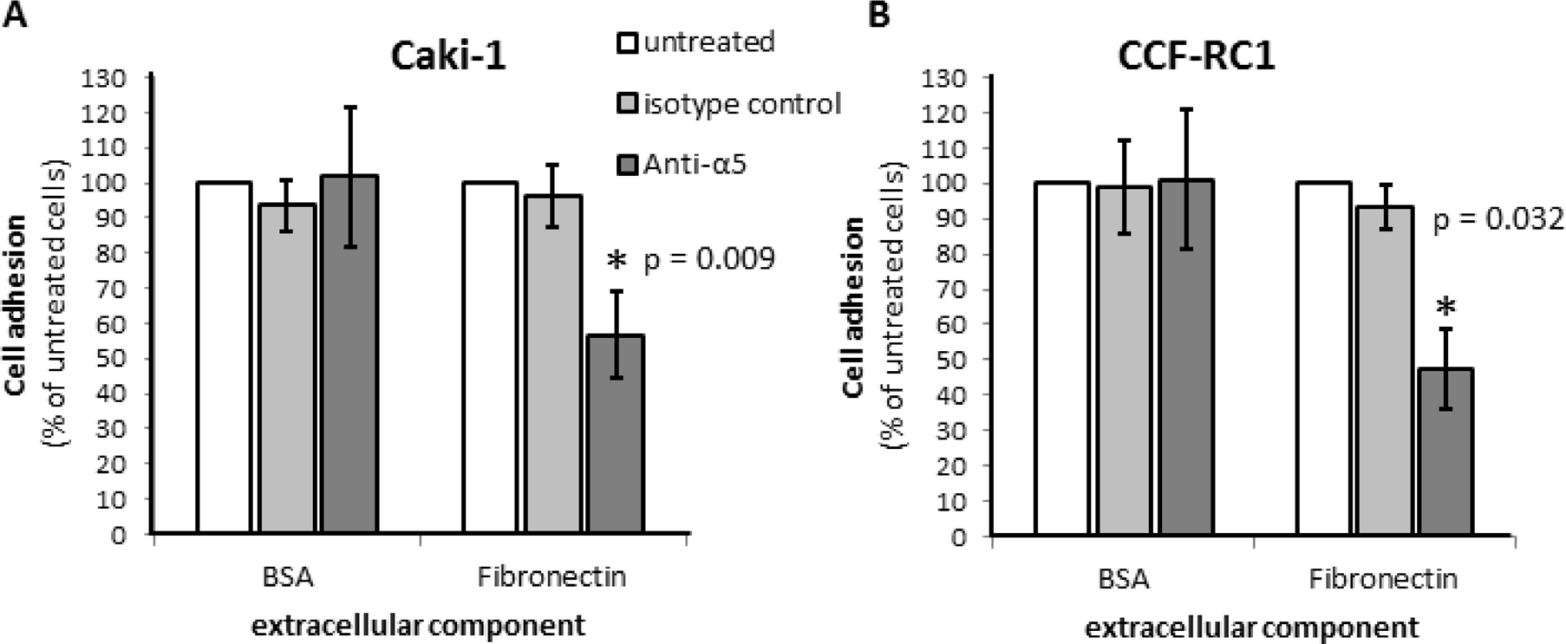
Cell adhesion of RCC cell lines Caki-1 (**A**) and CCF-RC1 (**B**) on extracellular matrix compound fibronectin. Cells were treated with an integrin α5 blocking antibody (Anti-a5, 10 μg/μl) or isotype control and cell adhesion on immobilized ECM compounds were determined. The adhesion value is shown as percentage of the adhesion of untreated cells. BSA was used as control. Integrin α5 blockade reduced cell adhesion to fibronectin significantly. Significance was calculated by Student‘s *T*-test, *p* < 0.05.

**Figure 7:**
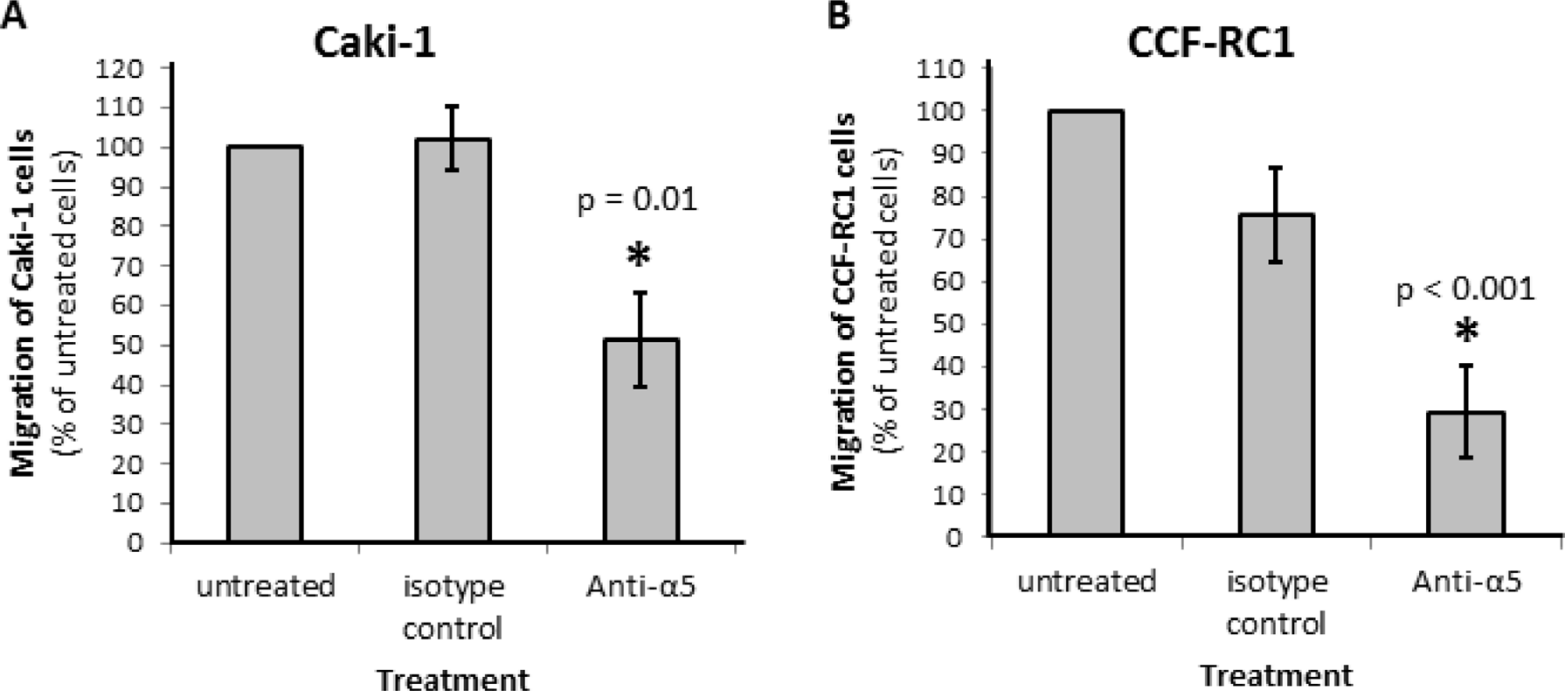
Chemotactical cell migration of RCC cell lines Caki-1 (**A**) and CCF-RC1 (**B**) using fibronectin as chemotaxin. Cells were treated with an integrin α5 blocking antibody (Anti-a5, 10 μg/μl) or isotype control. Migration was determined in a Boyden chamber using fibronectin (10 μg/ml) as chemotaxin. Integrin α5 blockade reduced cell migration significantly. Significance was calculated by Student‘s *T*-test, *p* < 0.05.

On the basis of the results in native tissue specimens we investigated the impact of integrin α5 on ccRCC progression *in vitro*. First, we analyzed the expression of integrin α5 in five ccRCC cell lines, CCF-RC1, 786-O, A498, Caki-1 and Caki-2, by flow cytometry. We found a strong expression of integrin α5 in Caki-1 and CCF-RC1 (Figure 5A, 5B). The integrin α5 expression of these two cell lines was immunohistochemically verified (Figure 5C). Afterwards Caki-1 and CCF-RC1 were treated with an anti-integrin α5 blocking antibody before cell adhesion to ECM, chemotactic migration and cell viability were analyzed. The chemotactic migration was determined in a Boyden chamber with the ECM component fibronectin as chemotactic agent. We selected fibronectin, because the integrin α5 subunit is associated with the integrin β1 subunit to form a fibronectin receptor [27] and in former investigations we could demonstrate a strong affinity for ccRCC cells to fibronectin [28]. The cell adhesion potential to fibronectin was decreased to 57% (p = 0.009) in Caki-1 and 47% (p = 0.032) in CCF-RC1 after integrin α5 blockade. There was no difference between isotype control and untreated cells (Figure 6). Integrin α5 blockade resulted in a decreased cell migration to 51% (p = 0.01) in Caki-1 and 30% (p < 0.001) in CCF-RC1 referred to untreated cells. Either a non-significant decrease of 24% in CCF-RC1 or no effect in Caki-1 cells was detected after treatment with an isotype control (Figure 7). Cell viability was determined by a cell metabolic assay was not influenced by integrin α5 blockade (data not shown).

### Influence of integrin α5 blockade on signaling pathways of RCC cells

**Figure 8:**
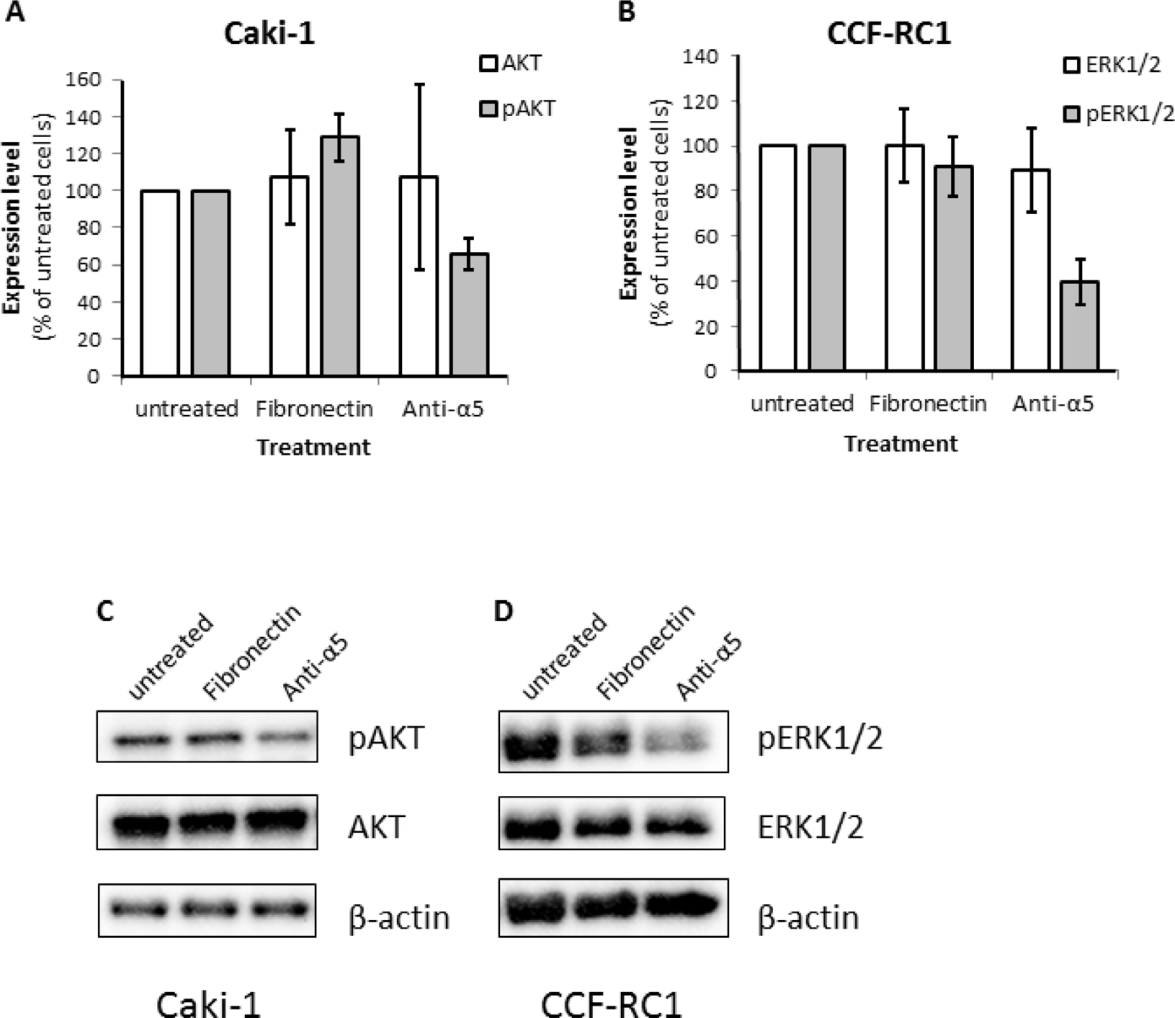
Expression level of relevant signaling molecules after blocking of integrin α5 (**A, C)** Expression and activity (phosphorylation status) of AKT (S473). (**B, D**) Expression and activity (phosphorylation status) level of ERK1/2 (ERK1 T202/Y204 and ERK2 T185/Y187). The RCC cells were treated with fibronectin (10 μg/ml) or an integrin α5 blocking antibody (Anti-α5, 10 μg/μl) antibody for 30 minutes.

To investigate the downstream signaling effects responsible for the reduced cell adhesion and chemotactic migration after blocking of integrin α5, we examined the expression and activity of the kinases FAK, ERK1/2, AKT and SRC as well as the adapter molecules Paxillin and SHC after blockade of integrin α5 or activation using fibronectin. In Caki-1 cells only AKT but not ERK1/2 showed a reduced phosphorylation status after integrin α5 blockade (Figure 8A, 8C). In contrast, in CCF-RC1 cells the kinase activity of ERK1/2 but not AKT was decreased after integrin α5 blockade compared to untreated or fibronectin-treated cells (Figure 8B, 8D). The expression or activity of all other analyzed signaling molecules was unchanged in both cell lines after integrin α5 blockade (data not shown).

### Activity of integrin α5 signaling pathway in renal tumor tissue and normal renal tissue

To analyze the impact of the downstream signaling molecules, we went back to the tumor samples and analyzed the expression and activity of ERK1/2, AKT, FAK as well as SHC in tumor specimens. Integrin α5 did not correlate with AKT or pAKT. In contrast, a correlation of integrin α5 levels with the amount of ERK1/2 (p = 0.052) was indicated, as well as with pERK1/2 (p = 0.058), pFAK (p = 0.049) and SHC. (p = 0.005, Table 2). These signal transducers correlated significantly with integrin α5 also in the corresponding normal renal tissues, generating p-values under 0.0001 for ERK1/2 and SHC and 0.049 for pFAK (Table 2). These results strengthen our *in vitro* findings and indicate that even the benign kidney tissues of RCC patients enable information about tumor parameters like tumor prognosis and progression.

**Table 2:** Correlation of the protein levels of the signaling molecules (p)ERK1/2, (p)AKT, pFAK (Y397) and SHC with integrin α5 expression in malign and benign tissue of patients with ccRCC

	tissue	*p*-value
ERK1/2	malign	0.052
	benign	< 0.0001
pERK1/2 (T202/Y204)	malign	0.058
	benign	0.465
AKT	malign	0.108
	benign	0.279
pAKT (S473)	malign	0.801
	benign	0.133
pFAK (Y397)	malign	0.049
	benign	0.049
SHC	malign	0.005
	benign	< 0.0001

Significance was calculated by a Spearman's Rho regression.

## DISCUSSION

Integrins, especially the subunit integrin α5, is known to be a potent trigger for tumor progression and metastasis in several tumor entities. In glioma cells, the expression and activation of integrin α5 is linked to an increase in migration [21]. Moreover, Jung *et al.* have shown that integrin α5 induces EMT and prometastatic properties in head neck squamous cell carcinoma tumors [29]. In renal cell carcinoma (RCC) Hase *et al.* [25] as well as our group [26] provided evidence for a progression promoting role of integrin α5 in kidney cancer, prompting us to analyze the physiological and molecular consequences of integrin α5 dosage in ccRCC.

In our study, integrin α5 was twice as high expressed in tumor tissues than in the corresponding benign renal tissues. Furthermore, in high-grade tumors it was significantly higher expressed than in low-grade tumors. This suggests that integrin α5 is involved in tumor development as well as in tumor progression of ccRCC. The correlation of the integrin α5 expression in RCC specimens with the gender of the patients strengthens this interpretation, since male patients show a higher integrin α5 expression and have a worse overall survival than female patients [30]. This observation is in good accordance with findings in other tumor entities like in esophageal squamous cell carcinoma where integrin α5 correlates with a worse overall survival and is associated with lymph node metastasis [31]. The prognostic relevance of integrin α5 level has also been demonstrated in ovarian cancer [23]. Similar results have been shown for non-small cell lung cancer and high-grade glioma [18, 19], suggesting that integrin α5 may have an oncogenic character.

The most important step of tumor progression is the development of metastases, frequently leading to patients’ death. During metastasis, tumor cells gain the ability to escape from the primary tumor, to enter into the circulation, extravasate into a distant niche and proliferate in the secondary organ. In the process of metastasis, cell migration and adhesion to ECM are essential steps. Therefore membrane-bound receptors recognizing ECM components like integrin α5 are expected to play a role in metastasis. In previous studies [26] we presented relevance of integrin α5 on bone-specific metastasis of renal cancer. In primary ccRCC cells obtained from patients who developed bone metastases within a period of five years after surgery, integrin α5 was significant higher expressed than in cells from patients without metastases [26]. In the present study we specified the impact of integrin α5 on metastasis by blocking integrin α5 on ccRCC cells and analyzing cell adhesion and chemotactic migration to fibronectin. In accordance with the situation in colon carcinoma cells [9], blocking of integrin α5 led to a decrease in cell adhesion of Caki-1 and CCF-RC1 cells and to a decrease of the chemotactic migration in direction to fibronectin. Cell viability, measured by metabolic cell activity, was not influenced by integrin α5 blockade, although integrins β1 have been found to regulate also the proliferation of ccRCC cells [32]. Our findings indicate a significant role of integrin α5 in the process of extravasation and invasion during metastasis of ccRCC cells but not in the proliferation within the metastatic niche.

Analyzing the activity of signaling pathways after integrin α5 inhibition in the two cell lines revealed ambivalent insignificant results with an activity decrease of ERK1/2 in CCF-RC1 cells and of AKT in Caki-1 cells. Furthermore an increase in AKT activity could be observed in Caki-1 cells after treatment with fibronectin. To establish the clinical relevance of these results and to specify the relevant signaling pathway *in vivo* we analyzed the integrin α5 expression and the activity of ERK and AKT in a cohort of 141 ccRCC specimens. Here we observed a correlation of integrin α5 with the activity of ERK1/2, but not of AKT, suggesting that the cell line CCF-RC1 reflects the *in vivo* situation more precise. Integrins are capable to activate ERK via FAK and SHC and consequently induce cell adhesion and migration [33]. In CCF-RC1 cells we already described the integrin β1-FAK axis to be responsible for cell migration [34]. Analyzing FAK and SHC in the ccRCC cohort confirmed the impact of this signaling pathway, since the activity of SHC and FAK also correlated with the integrin α5 expression. From our results we can conclude that metastasis of ccRCC is mediated by integrin α5, activating the ERK signaling pathway via SHC and FAK.

Targeting integrin α5 in ccRCC patients with a high expression level may be a promising strategy for individual therapy. This idea is supported by the finding that the integrin α5 blocking antibody volociximab showed promising results in cancer treatment *in vivo* in rabbits with Vx2 tumors [35, 36]. Initial phase I and phase II studies indicate a relatively good tolerance of this substance [37, 38]. As alternative to blocking antibodies also non-RGD-based peptide inhibitors like ATN-161 are under development. For ATN-161 a reduced tumor progression was observed in breast and colon cancer *in vivo* [39, 40], and a phase I study demonstrated a stable disease in one-third of all patients with solid tumors [41]. Our results suggest that also renal cancers may be suitable for an anti-integrin α5 therapy likewise.

Our analyses of normal renal tissue specimens obtained from ccRCC patients showed a significant correlation between integrin α5 expression and both, the development of metastases and patients’ death. Interestingly, in tumor tissue specimens this correlation was missing. This discrepancy may be caused by a large composition of cellular factors in tumor cells influencing the metastatic behavior and consequently resulting in a larger deviation in these specimens. The finding that the integrin α5 expression in normal renal tissue correlates with tumor progression however suggests that integrin α5 is suitable as a prognostic factor in the healthy kidney of ccRCC patients. This may be caused by the circumstance that either the progress of the tumor is determined by the integrin setting of the normal renal tissue or the benign tissue is influenced by the tumor. Similarly, Joeckel *et al.* showed that the expression of the Calcium-sensing receptor (CaSR) in the normal renal tissue determines the probability of developing bone metastases in ccRCCs [42]. The use of integrin α5 expression in benign tissue as a prognostic biomarker for ccRCC has great advantages. Although it is generally accepted that obtaining a biopsy from RCC is a highly safe procedure [43, 44] and this approach has the advantage of getting information about histology and grading of the tumor, some colleagues still implicate a risk to cause dissemination of tumor cells through the lesion [45], what may hamper the consent of the patients. This problem could be avoided if usable prognostic information can be obtained from biopsies of normal renal tissue.

## CONCLUSIONS

Our study shows that a higher integrin α5 expression level in kidneys seems to cause a worse outcome for patients with ccRCC. This might be induced by an ERK mediated increased migration potential as well as a higher adhesion of the tumor cells to fibronectin, both important aspects for tumor progression and development of metastases. Therefore, integrin α5 could potentially play a prominent role as prognostic marker for the RCC, determinable not only in tumor tissue but also in normal renal tissue. A specific inhibition of integrin α5 or its downstream targets might be useful approaches for the targeted therapy of RCC leading to better patients’ prognosis and individual therapy options.

## MATERIALS AND METHODS

### Specimens

Tissue samples were obtained under sterile conditions from 141 patients with primary RCC (Table 1) who underwent nephrectomy at the Department of Urology, University Medical Center Mainz [46]. The study was performed in agreement with the Declaration of Helsinki and approved by local ethics committee (No. 837.005.09, Landesärztekammer Rheinland-Pfalz, Mainz, Germany). Each patient provided informed consent. Samples of tumor tissue and renal cortex, obtained from the opposite kidney pole at a minimum of three cm from the tumor were shock frozen in liquid nitrogen and stored at –80°C for at least five years. The diagnosis of RCC was verified on hematoxylin and eosin sections and the tumor grade determined.

### Cells and cell culture

The human renal cell carcinoma cell lines A498, 786-O, Caki-1 and Caki-2 were obtained from LGC Promochem (Wesel, Germany) and CCF-RC1 was kindly provided by the establisher, Cleveland Clinic Foundation [47]. A498 has been generated from a 52 year old female, 786-O from a 58 years old male patient. These two cell lines have a VHL deletion. Caki-1 has been isolated from a skin metastasis of a 49 years old male, Caki-2 from a RCC of a 69 years old male patient. These tow cell lines have no VHL mutation or deletion. CCF-RC1 was isolated from a high grade clear cell RCC of a 67 years old male with bone metastases, which had invaded in the perirenal fatty tissue [47]. All cell lines are assigned to clear cell RCC, although A498 and Caki-2 cells are also discussed to be from papillary RCC [48]. Caki-1 and Caki-2 cells were cultured in Iscove's (Biochrom), supplemented with 10% fetal calf serum, 1% GlutaMax (Sigma) and 1× penicillin/streptomycin (Anti/Anti 100×; Life Technology). CCF-RC1, 786-O and A498 cells were cultured in RPMI 1640 (Gibco) supplemented with 10% fetal calf serum, 2.5% HEPES buffer (Sigma) and 1× penicillin/streptomycin (Life Technology). All cell lines were incubated in a moistened atmosphere at 5% CO_2_ at 37°C in air.

### Flow cytometric analysis of integrin a5 in renal carcinoma cell lines

The cell lines Caki-1, Caki-2, CCF-RC1, 786-O and A498 were analyzed for integrin α5 protein levels. Cells were detached by using trypsin-EDTA, since an EDTA detachment without trypsin only causes a marginal cell protection (data not shown). After centrifugation at 300 g cells were resuspended in 10 ml DPBS and twice centrifuged for five minutes at 300 g. Tumor cell suspension (0.5 × 10^6^ cells) was transferred in flow cytometric analyzing tubes and centrifuged for five minutes at 300 g. The supernatant was removed. The remaining cell pellet was resuspended in 100 μl DPBS + 1% BSA. Cells were treated with an isotype control PPV-07 antibody, an IgG3 monoclonal antibody mouse type (1:10 DPBS + 1% BSA, abcam) or with P1D6 antibody, an anti-integrin α5 antibody (1:100 DBPS + 1% BSA, abcam) for 30 min on ice. After washing with DPBS cells were incubated with 100 μl secondary antibody (AlexaFluor^®^ 488 rabbit anti-mouse IgG (H+L) 1:10 DBPS + 1% BSA, lifetechnologies) for 30 minutes on ice in darkness. Cells were washed by using DBPS and centrifugation (300 g, five minutes), the supernatant discarded and the remaining cell pellet was resuspended in 500 μl DPBS + 1% BSA for analysis. 15000 counts were used for analysis and interpreted on behalf of integrin α5 protein level.

### Immunohistochemical staining of integrin α5 in Caki-1 and CCF-RC1 cells

Cells were transferred on microscope slides using a cytospin centrifuge. For immunohistochemical staining DAKO EnvisionFlex^®^ Minikit K5023 was used. Cells were fixed on microscope slides with 100% ethanol for 10 minutes and washed with H_2_O. Endogenous peroxidase was blocked using peroxidase blocking solution for 10 minutes. Slides were then incubated with primary P1D6 anti-integrin α5 antibody (1:200) in antibody diluent for one hour at room temperature. After washing the microscope slides three times with washing buffer, slides were incubated with 100 μl visualization reagent for 30 minutes at room temperature. Subsequently 100 μl DAB-reagent (1 ml DAB substrate buffer plus two drops of DAB chromogen) was added on the slides and incubated for five minutes. Finally staining with hematoxylin was performed for one minute, followed by 10 minutes incubation in an increasing alcohol row and a decreasing xylol row (each concentration for three minutes). Stained slides were then analyzed at 20-fold and 40-fold amplification by microscopic analysis.

### Cell adhesion assay

For cell adhesion assay, amine-binding, maleic anhydride activated clear 96-well-plates (Pierce #15110, Thermo Scientific) were used. Each well was coated with extracellular matrix components at a volume of 100 μl overnight on a rocking shaker at room temperature. Components used were fibronectin (10 μg/ml) and as control BSA (10 μg/ml). On the next day, wells were washed twice with 100 μl washing buffer (DPBS with 0.05% Tween 20, ICI Amenic Inc.). Unspecific binding sites were blocked with 200 μl blocking solution (DPBS with 0.5% BSA) per well and incubated for one hour in a moistened atmosphere at 5% CO_2_ at 37°C in air. Meanwhile cells were washed in DPBS, detached with Trypsin-EDTA, resuspended in serum-free culture medium and treated for 30 minutes with anti-integrin α5 antibody (P1D6 10 μl/ml, abcam) or an anti-HLA class 1 isotype control EMR8-5 (10 μl/ml, abcam), respectively. Blocking solution was removed and 50 μl of tumor cell suspension (4 × 10^5^ cells/ml) per well were added. After one hour incubation in a moistened atmosphere at 5% CO_2_ and 37°C, non-adherent cells were washed out with 2 × 200 μl washing buffer per well. Adherent cells were fixed with 100 μl 4% paraformaldehyde (Histofix 4%, Roth) per well for 15 minutes at room temperature. Fixation medium was washed out with 100 μl DPBS. Adherent and fixed cells were stained using 100 μl crystal violet solution (5 mg/ml in 2% ethanol) for 10 minutes at room temperature. Afterwards the staining solution was washed out three times with 100 μl washing buffer per well and the plate was air-dried. For resolving the colorant wells were incubated with 100 μl 2% SDS (Roth) for 30 minutes on a rocking shaker. The absorbance was measured at 550 nm (reference value at 650 nm) with GloMax^®^-Multi detection system (Promega) [26]. Experiments were performed in quadruplicates and repeated three times. Mean value and standard error rate were calculated. For normalization between treated and untreated cells percentage of adherent cells was used, setting adhesion of untreated cells at 100%.

### Chemotactic cell migration assay

For chemotactic cell migration analyses a modified Boyden chamber was used (Costar) [42]. The chamber consisted of 48 wells (each 3.17 mm^2^) which are divided in an upper and a lower compartment separated by a porous polycarbonate membrane with 8 μm pore diameter (Neuro Probe). Before analysis cells were cultivated in serum-free culture medium for 24 hours. According to the construction of the manufacturer, the lower chemotaxis compartment was filled with 29 μl solution of extracellular matrix components (fibronectin 10 μg/ml [28], Thermo Scientific) diluted in serum-free medium. The lower part was then covered by the polycarbonate membrane, which was equilibrated before in DPBS for two minutes. The membrane was then covered by the upper part and fixed. The wells of the upper part were filled with 50 μl of tumor cell suspension (3 × 10^5^ cells/ml). Cells were pre-treated for 30 minutes with 10 μg/ml anti-integrin α5 antibody (P1D6, abcam), an isotype control (EMR8-5, abcam), or untreated for comparison. The chamber was then incubated for 16 hours in a moistened atmosphere with 5% CO_2_ at 37°C. Afterwards non-migrated cells were removed from the upper membrane side by washing it in buffer solution according to Weise (Merck) and by mechanical detachment using a rubber scraper. The polycarbonate membrane was dried and migrated cells were fixed for one minute in methanol. Cytoplasm and cell nuclei were dyed with hemacolor (Merck). The dyed membrane was put onto a microscope slide and covered with immersion oil. The migrated cells were counted on an area of 2.5 mm^2^ of the porous membrane. The experiment was performed in quadruplicates and repeated three times. For statistical analysis mean value and standard error rate were calculated. For comparison between treated and untreated cells the percentage of migrated cells was used, setting migration of untreated cells towards fibronectin at 100%.

### Cell viability assay

Cell viability was studied using CellTiter-Glo^®^ Luminescent Cell Viability Assay (Promega) according to manufacturer's protocol. Cells were seeded in a 96-well-plate (5 × 10^3^ cells per well) in quadruplicate. Cells of the intervention group were after 30 minutes treated with 10 μg/ml P1D6 anti-integrin α5 antibody and with 10 μg/ml fibronectin after one hour. The positive control was only treated with equal fibronectin concentration after one hour. Both groups were compared to untreated cells. Luminescence was detected every 30 minutes using GloMax^®^ Multidetection System (Promega) for a total of eight measurements. Relative luminescence units were plotted versus time on x-scale.

### Western blot analysis

For preparation of protein extracts from renal tissue a 50 mg piece was transferred in a reaction tube with 1 ml lysis buffer (2 mM HEPES, 0.02 M NaCl, 0.05 mM MgCl_2_, 0.04 mM EDTA, 0.1% Triton X-100, 5 μM DTT, 1% Phosphatase Inhibitor Cocktail II (Sigma), 1% Protease Inhibitor Cocktail (Sigma)) [49]. Afterwards the tissue specimen was shredded twice with ultrasound. For preparation of protein extracts from cell culture, tumor cells (7.5 × 10^5^ cells) were seeded on 100 mm^2^ cell culture plates. On the day before protein extraction, culture plates were put on serum-free culture medium. For protein extraction, cells were washed with DPBS and mechanically detracted in lysis buffer with a cell scraper, transferred in a 2 ml reaction tube and placed on ice. After incubation for 30 minutes the samples were centrifuged for 10 minutes at 14000 g. The supernatant was transferred in a new tube and stored at –20°C. For evaluating protein concentrations of the extracts, BCA-reagents (Pierce BCA Protein assay kit, Thermo Scientific) were used. For protein precipitation 9-fold volume of acetone was added.

Equal amounts of protein (50 μg per lane) were size-separated by SDS-PAGE with 10% or 7.5% polyacrylamide gels. Afterwards gels were transferred on PVDF membrane by semidry blotting. Membranes were blocked according to instruction manual of antibody manufacturer for one hour. Next, membranes were incubated with primary antibody in blocking solution overnight on a roll mixer at 4°C. The monoclonal mouse antibodies against extracellular-signal regulated kinases 1/2 (ERK1/2) (R&D) and rabbit antibodies against phospho-ERK T202/Y204 (R&D), integrin α5, FAK, phospho-FAK Y397, Paxillin, phospho-Paxillin Y118, SHC, SRC, phospho-SRC Y416, AKT, phospho-AKT S473 and T308 (all CST) were used at a dilution of 1:1000. β-actin antibody (Sigma) was employed at a dilution of 1:5000. After washing the membrane three times for 10 min it was incubated with HRPlinked secondary antibodies (DAKO) at a dilution of 1:1000 for one hour at room temperature. The membrane was washed again and bound antibodies were visualized by adding enhanced chemiluminescent solution (Perkin/Elmer). A chemiluminescent detector (FluorChemE, Protein Simple) was used for imaging. For quantification a computer-based pixel counting system was used (AlphaView, Protein Simple), subtracting the background from the visual band. These values were normalized to β-actin amounts (for cell extracts) or Coomassie staining (for tissue specimen) values of the same membrane as loading control. Untreated cells were compared to cells treated with fibronectin only or treated with fibronectin and anti-integrin α5 blocking antibody (P1D6, ab78614, Abcam) and an isotype control (EMR8-5, abcam). The cell culture experiments were performed three times. Mean value and standard error rate were calculated.

### Statistical analysis

For statistical analysis we used IBM-SPSS 22.0 and Microsoft Excel 2013. Signaling molecules expression results were quantified and presented as relative units. Significances of tissue specimen analyses were calculated by using the Mann–Whitney *U*-test in relation to patient's gender, grading, metastasis and 5-year-survival. For analysis of differences in the total expression the Wilcoxon signed-rank test was performed. Regression analyses were performed using a Spearman's Rho test and a Cox regression. All other results using RCC cell lines were calculated as % of untreated cells. Differences in expression levels, adhesion and migration potential were determined using the Student's *T*-test. Statistical significance was assumed at a *p*-value of < 0.05.

## Abbreviations

AKT: AKT8 virus oncogene cellular homolog
CaSR: calcium-sensing receptor
ccRCC: clear cell RCC
CEA: carcinoembryonic antigen
ECM: extracellular matrix
ERK: extracellular signal-regulated kinase
FAK: focal adhesion kinase
MAPK: mitogen activated protein kinase
MMP: matrix metalloproteinase
PI3K: phosphatidylinositol-4,5-bisphosphate 3-kinase
RCC: renal cell carcinoma
SFK: SRC-family kinase
SHC: SRC homology 2 containing-protein
SRC: rous sarcoma oncogene homologue
